# Current state of rare disease registries and databases in Australia: a scoping review

**DOI:** 10.1186/s13023-023-02823-1

**Published:** 2023-07-27

**Authors:** Rasa Ruseckaite, Chethana Mudunna, Marisa Caruso, Falak Helwani, Nicole Millis, Paul Lacaze, Susannah Ahern

**Affiliations:** 1 Department of Epidemiology and Preventive Medicine, Monash University, Melbourne, VIC 3004 Australia; 2 Rare Voices Australia, VIC 3194, Melbourne, Australia

**Keywords:** Rare disease, Minimum data set, Registry, Patient outcomes, National approach

## Abstract

**Background:**

Rare diseases (RDs) affect approximately 8% of all people or > 400 million people globally. The Australian Government’s National Strategic Action Plan for Rare Diseases has identified the need for a national, coordinated, and systematic approach to the collection and use of RD data, including registries. Rare disease registries (RDRs) are established for epidemiological, quality improvement and research purposes, and they are critical infrastructure for clinical trials. The aim of this scoping review was to review literature on the current state of RDRs in Australia; to describe how they are funded; what data they collect; and their impact on patient outcomes.

**Methods:**

We conducted a literature search on MEDLINE, EMBASE, CINAHL and PsychINFO databases, in addition to Google Scholar and grey literature. Dissertations, government reports, randomised control trials, conference proceedings, conference posters and meeting abstracts were also included. Articles were excluded if they did not discuss RDs or if they were written in a language other than English. Studies were assessed on demographic and clinical patient characteristics, procedure or treatment type and health-related quality of life captured by RDRs or databases that have been established to date.

**Results:**

Seventy-four RDRs were identified; 19 were global registries in which Australians participated, 24 were Australian-only registries, 10 were Australia and New Zealand based, and five were Australian jurisdiction-based registries. Sixteen “umbrella” registries collected data on several different conditions, which included some RDs, and thirteen RDRs stored rare cancer-specific information. Most RDRs and databases captured similar types of information related to patient characteristics, comorbidities and other clinical features, procedure or treatment type and health-related quality of life measures. We found considerable heterogeneity among existing RDRs in Australia, especially with regards to data collection, scope and quality of registries, suggesting a national coordinated approach to RDRs is required.

**Conclusion:**

This scoping review highlights the current state of Australian RDRs, identifying several important gaps and opportunities for improvement through national coordination and increased investment.

## Background

Rare diseases (RDs), by definition, affect fewer than five in 10,000 people [1, 2]. Estimates of the total number of RDs vary between countries and studies, due to differing definitions and challenges with data collection. However, it is cited that there are more than 7000 different RDs [3]. Approximately 80% of RDs are of genetic origin. As genomic technology evolves, new RDs are discovered regularly [1]. Although individual RDs are rare, the total number of Australians living with a RD is sizeable. Approximately 8% of Australians live with a RD, equating to around two million people [4].

RDs often manifest in childhood and become chronic; some are life threatening and others lead to significant disability [5, 6]. Diagnosis of RDs is often delayed, due to limited knowledge, lack of exposure to and awareness of healthcare professionals to RDs and uncertainty about referral pathways. Inherent features of RDs, including heterogeneity, complexity and low patient numbers result in a lack of data, evidence and knowledge [1], making data collection and registries critical for RDs.

Rare disease registries (RDR) are established to collect RD data and in some cases, monitor clinical outcomes [1, 7]. Registries, if populated with high-quality clinical data over extended periods of time, can assist with health service planning, epidemiological research, clinical trial recruitment and post-marketing drug surveillance [1, 8]. RDRs can play a vital role in understanding disease trajectories and help developing clinical trials for RD that meet safety and efficacy criteria despite low patient numbers; with many recruiting fewer than 100 patients worldwide [9].

The critical role of RDRs is globally recognised by the RD community and policy makers [1, 6, 10]. International experts jointly identified ten key principles of RDRs, including: the need for RDRs to be a global priority; the importance of scope and focus; interoperability and harmonisation; consistency through minimum core data elements; links with biobank data; inclusion of patient reported data; sustainability; and governance and building knowledge [11].

Nonetheless, in Australia, data for most RDs are not being captured through routine health information systems or registries, and there is no coordinated strategy to collect, measure, build and translate already existing information on RDs [1].

On behalf of the Australian Government, Rare Voices Australia (RVA), the national peak body for Australians living with a RD [12], led the collaborative development of the National Strategic Action Plan for Rare Diseases (the Action Plan) [13], which was informed by extensive multi-stakeholder consultation. The Action Plan was launched in February 2020 with bipartisan support and RVA is now leading its collaborative implementation. The Action Plan called for the development of a national, person-centred approach to RDRs to support national standards, best practice and minimum data sets. It further highlighted the need for investment into RDRs, which aligns with worldwide agreement on the value and importance of RDRs [13].

Under Pillar 3, Research and Data, of the Action Plan, Priority 3.1 calls on the sector to ‘Enable coordinated and collaborative data collection to facilitate the monitoring and cumulative knowledge of rare diseases, informing care management, research and health system planning’ [13]. The first step to this end is outlined in Implementation step 3.1.4.1, ‘Develop a summary report of all existing Australian and relevant international rare disease registries’ [13], which provided the impetus for this scoping review.

This scoping review investigates RDRs collecting Australian data in the literature, including an audit of what RDRs and databases exist in Australia, how they are funded, what data they collect and their impact on patient outcomes. It is the first step to understanding existing datasets and informing national coordination of RDRs that aligns with international best practice for RD data collection, including establishment of minimum data sets. A national approach to RDR’s in Australia would enable improved monitoring and the accumulation of knowledge about RDs to inform clinical practice, research, government investment in RD and health service planning [14]. Data collected should also inform planning and investment decisions for other government services and departments playing an essential role in the holistic care of patients, carers and families living with rare disease including, the National Disability Insurance Agency, Department of Communities and Housing, Department of Education, and other financial support services.

## Methods

### Protocol

A protocol for this scoping review follows the Preferred Reporting Items for Systematic Reviews and Meta-Analyses extension for scoping reviews (PRISMA-ScR) format [15].

### Information sources

To identify relevant studies, we searched four databases: MEDLINE, EMBASE, CINAHL and PsychINFO, and the Google Scholar from inception through November 2022. Grey literature was also included. For each article selected for inclusion, abstracts and full articles were obtained. Reference lists of the included studies and systematic reviews were examined during the review. To capture as many relevant studies as possible, we did not establish a time frame for when articles were published. Dissertations, government reports, randomised control trials, conference proceedings, conference posters and meeting abstracts were included.

### Search strategy

The search strategy was developed by three researchers (RR, MC and CM). We used Medical Subject Heading (MeSH) keywords and free text search terms. The most up to date version of Chrome Web browser was used in our searches. The database records and details of how the literature search was undertaken were maintained at each stage of the review process. Also, a manual search using Google and Google Scholar was performed. The key search terms were ([“rare” or “disease” or “rare diseases” or rare adj disease* or condition* or disorder* or illness* or infection* or diagnosis*] and [“genetic” or “genetic, population” or “genetics, medical” or “genetics, microbial” or “human genetics”] and [“registry” or “registries” or “database” or “dataset” or “library” or “record” or “archive”] and [“Australia” or “Victoria” or “New South Wales” or “South Australia” or “Western Australia” or “Tasmania” or “Queensland” or “Northern Territory” or “Melbourne” or “Sydney” or “Adelaide” or “Canberra” or “Hobart” or “Brisbane” or “Darwin” or “Perth”]). We adapted the search strategy to the search requirements of the remaining databases mentioned above. The terms were combined by means of Boolean operators.

### Eligibility criteria

Quantitative and qualitative studies describing existing RDs and RDRs were included. International studies without Australian data, were excluded from this review, as they do not provide information on Australia’s RDR landscape. Furthermore, studies of diseases that are not rare were also excluded. Articles were excluded if they did not discuss a RD or if they were written in a language other than English.

### Study selection

The study selection process was made up of two phases. In phase one, two researchers conducted the initial search of the literature (RR and CM). The second phase consisted of screening the literature, where three researchers (RR, MC and CM) independently screened the titles and abstracts of all articles identified through the search strategy to determine eligibility and classify studies as relevant, possibly relevant and irrelevant. Results were discussed by all researchers to resolve any inconsistencies in study selection and a final list of relevant studies were made.

### Data management and analysis

The results from the database searches were extracted into EndNote™ X9 software, a management software for references that allows the identified references to be organized into different electronic databases. The results were combined into a single EndNote folder. Duplicated studies were identified and removed.

Data were extracted from relevant articles and internet material using a standardised data extraction form in Microsoft Excel. The main data points extracted include: objectives of the registry, details of registry management, population size, data captured by the registry, demographic or clinical or patient-reported outcome measures (PROMs) and quality of life (QoL) data, funding information, ethics and consent models of the registry.

## Results

### General description of the literature

**Fig. 1:**
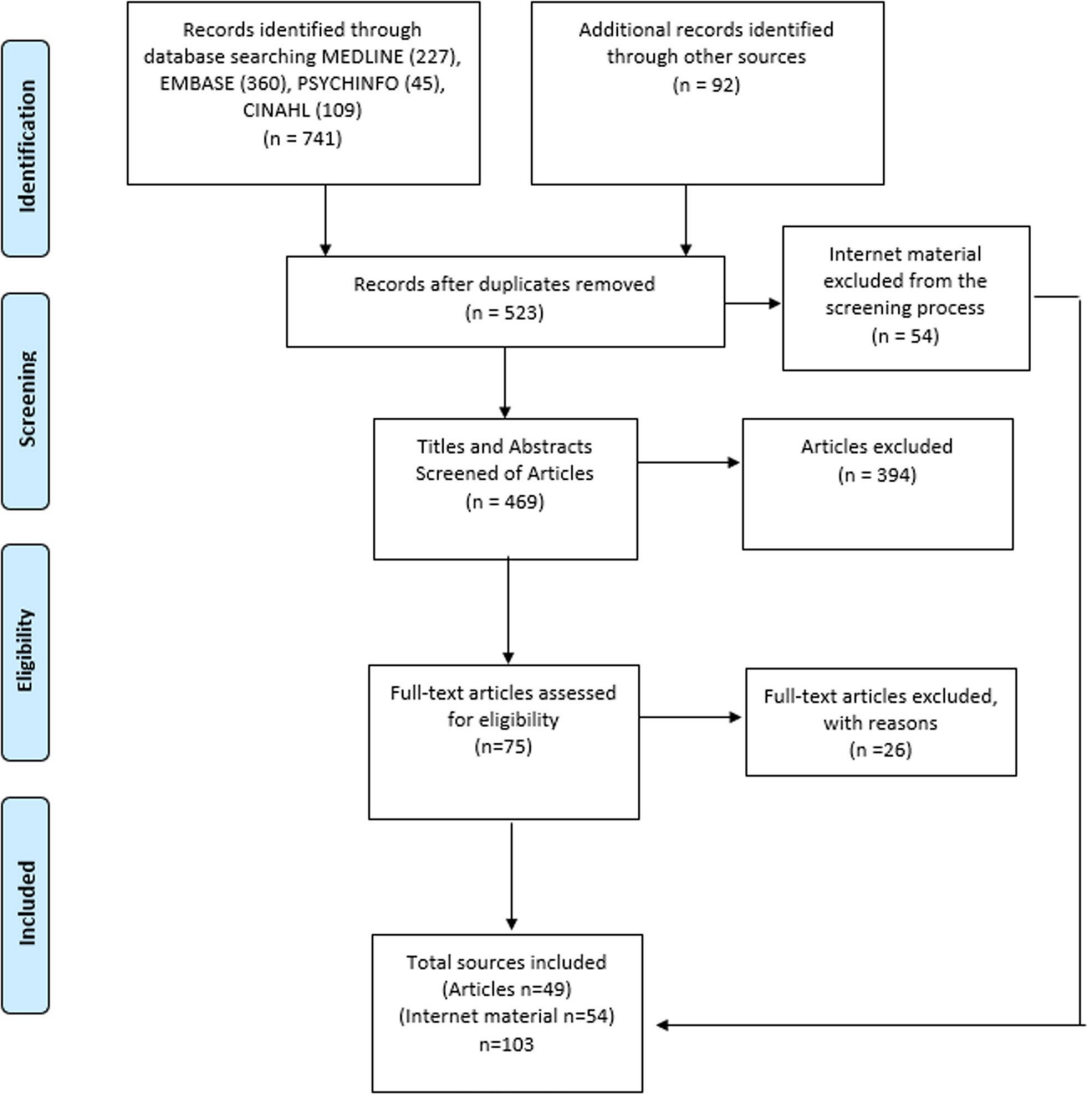
Study flow. Details the flow of information through the different phases of the review; maps out the number of records identified, included and excluded

The search of MEDLINE, EMBASE, CINAHL and PsycINFO databases yielded in 741 documents (Fig. 1). A further 92 documents of grey literature were identified. After removing duplicates, 523 documents were screened for titles and abstracts. Of those, full copies of 75 relevant articles were retrieved. Fifty-four online publications (e.g. webpages and reports) of Australian RDRs and online databases were included. The screening of full-texts resulted in 49 publications. A total of 103 publications and online data sources were used in the data extraction and data analysis process.

Publications included in the final review were published between 2002 and 2021. Five (10.2%) articles were published in 2017 and six (12.2%) were published in 2019, 2020 and 2021. The remaining articles were published between the 2002 and 2016.

Of the 49 publications used in the scoping review, 20 (40.8%) were published in Australia, seven (14.3%) were published in the United States (US), six (12.2%) in the Netherlands, two (4.1%) in the United Kingdom (UK) and one each (2.04%) was published in Japan and Canada.

### Rare disease registries

Seventy-four different RDRs and databases were described in these studies (Table 1). Nineteen (25.7%) of those were global registries that included Australian data, 24 (32.4%) were Australian national registries and covered all jurisdictions, 10 (13.5%) were Australian and New Zealand (ANZ) and five (6.8%) were Australian state and jurisdiction-based registries. Sixteen (21.6%) “umbrella” registries, which represent numerous registries that have agreed to work under a unified registry, collected data from several RDs or conditions, which included some RDs. Thirteen (17.6%) registries stored data that were specific to rare forms of cancer.

**Table 1:** General description and characteristics of rare disease registries, databases and organisations identified in the scoping review

Name	Size	Year established	Type	Patient/clinician; single/multicentre	Data entry method	Reporting	Funding source	Consent model
International Collaborative Gaucher Group Gaucher Registry	12,000 participants	1994	Global	Clinician; Multicentre	Web-based data entry	NS	Genzyme	Opt in
Global Retrospective Registry for Danon Disease	82 patients (2021)	2006	Global	Clinician; Multicentre	E-mail & telephone	NS	NS	NS
Global Registry of Acute Coronary Events	10,2000 patients (2021)	1999–2009	Global	Clinician; Multicentre	NS	NS	NS	Opt in
International & SIOP-E Diffuse Intrinsic Pontine Glioma (DIPG) Registries	Estimated > 1000 patients (2020)	2011	Global	Clinician; Multicentre	Online web application & database	Annual report	The DIPG Collaborative	Consent required
International Dysferlinopathy Registry	NS	2012	Global	Clinician; Multicentre	Online	NS	The Jain Foundation	Consent required
International Schwannomatosis Database	389 patients (2017)	2011	Global	Clinician; Multicentre	Online	NS	Industry	Consent required
International Pachyonychia Congenita Research Registry	2000 patients (2018)	2004	Global	Patient; Multicentre	Web-based data entry	NS	Pachyonychia Congenita Project Non-profit Organization	Consent required
Wolfram Syndrome International Registry	21 patients (2009)	2009	Global	Patient; Multicentre	NS	NS	NS	Consent required
The Global Fukutin Related Protein Registry	622 patients (2018)	2011	Global	Patient; Multicentre	Web-based data entry	Newsletters	LGMD2iFund & CureLGMD2i	Voluntary opt in/opt out
Cantú Syndrome Registry	74 (2019)	2012	Global	Patient; Multicentre	Paper form, electronic form through REDCap	Newsletters	NS	Consent & recruitment procedures vary
GNE Myopathy Registry	432 patients (2021)	2014	Global	Patient; Multicentre	Online	Newsletters	Translational Research in Europe-Assessment & Treatment of Neuromuscular Diseases network	Electronic consent
Global Prader-Willi Syndrome Registry	1696 participants (2019)	2015	Global	Patient; Multicentre	Online	NS	Foundation for Prader-Willi Research	Opt in
Global Angelman Syndrome Registry	> 1300 families	2016	Global	Patient; Multicentre	Online database	NS	Foundation for Angelman Syndrome Therapeutics AUS	Consent required
Idiopathic Thrombocytopenic Purpura Natural History Study Registry	1448 patients (2021)	2017	Global	Patient; Multicentre	Web-based data entry	Annual reports & fundraisers	Platelet Disorder Support Association	Voluntary participation
Friedreich’s Ataxia Global Patient Registry	1000+ patients (2021)	2019	Global	Patient; Multicentre	Online	Australian annual reports; newsletters	Friedreich’s Ataxia Research Alliance	Consent required
The Myotubular & Centronuclear Myopathy Patient Registry	228 patients (2018)	2013	Global	Patient; Multicentre	Online	Newsletters	Myotubular Trust, Muscular Dystrophy UK & Audentes Therapeutics	Opt in
International Niemann-Pick Disease Registry	1000 patients	2013	Global	Patient; Multicentre	Web-based data entry	Annual reports; newsletters	Consumers, Health, Agriculture & Food Executive Agency grant	Consent required
Limb-Girdle Muscular Dystrophy Type 2A Patient Registry	NS	NS	Global	Patient; Multicentre	Online	NS	Fundraisers	NS
International Fragile X Premutation Registry	> 2300 patients (2014)	2009	Global	Patient; Multicentre	Online	NS	CDC	Consent required
Australian & New Zealand Fontan Registry	1682 patients	2009	ANZ	Clinician; Multicentre	Web-based data entry	Annual report, registry publications	Heart foundation, MCRI, ANZ trustees, Heart Kids, Australian Government	Opt out
Thrombotic Microangiopathies Registry	340 patients (2020)	2009	ANZ	Clinician; Multicentre	Online REDCap database	Newsletter	Monash University (In kind)	NS
Australasian Epidermolysis Bullosa Registry	417 patients (2012)	2006	ANZ	Clinician; Multicentre	Online database	Research publications	The Dystrophic Epidermolysis Bullosa Research Association of Australia	Opt in
Lymphoma & Related Diseases Registry	4500 patients (2021)	2016	ANZ	Clinician; Multicentre	Web-based data entry	NS	Industry partners	Opt out
Myeloma & Related Diseases Registry	4747 patients (2021)	2012	ANZ	Clinician; Multicentre	Web-based data entry, Paper QoL	Research publications, annual report, 6-monthly reports to sites, newsletter	Industry partners, Myeloma Australia	Opt out
Australia & New Zealand Transplant Registry*	29,997 patients (2021)	1977	ANZ	Clinician; Multicentre	Online or postal	Annual report	Government, Industry	Opt out
Australian & New Zealand Registry of Advanced Glaucoma*	> 2000	2007	ANZ	Clinician; Multicentre	Paper & online	Newsletter	Government, Industry	NS
Australasian Interstitial Lung Disease Registry	> 2000	2016 (Australia)	ANZ	Clinician; Multicentre	Retrospective data entered at 1^st^ clinic visit with prospective data—after each subsequent visit	NS	Lung Foundation	Consent required
The Australasian Registry Network of Orphan Lung Diseases	186 patients	Jul 2009 & Jun2014	ANZ	Clinician; Multicentre	NS	Publication	Lung Foundation & Mr Ivan Cash	Opt in
Australia & New Zealand Familial Hypercholesterolaemia Registry	1500 patients (2021)	2015	ANZ	Clinician; Multicentre	EMR	Annual report	NS	Opt in
Australian National Creutzfeldt-Jakob Disease Registry	1321 cases (2019)	1993	AUS	Clinician; Multicentre	Forms completed by clinicians, families	Annual Reports	Government	Opt in
The Australian Rett Syndrome Database	311 patients (2007)	1993	AUS	Clinician; Multicentre	Family & clinical questionnaire	Newsletter	New South Wales Rett Syndrome Association & Rett Syndrome Australian Research Fund	Opt in
Lymphangioleiomyomatosis Registry	100 patients (2014)	2006	AUS	Clinician; Multicentre	NS	Newsletter	Not-for-profit charity	NS
Australian Genetic Heart Disease Registry	1600 patients (2013)	2007	AUS	Patient, Multicentre	Online database	Bi annual newsletter	Agnes & Berel Gings, Pfizer, Boston Scientific, Cardiomyopathy Association of Australia	Opt in
Neonatal Alloimmune Thrombocytopenia Registry	76 patients (2015)	2009	AUS	Clinician; Multicentre	Online database	Periodic newsletters	Monash University's Department of Epidemiology & Preventative Medicine	Opt out
Australian Idiopathic Pulmonary Fibrosis Registry	647 patients (2017)	2012	AUS	Clinician; Multicentre	Web-based data entry	Newsletters	Educational grants & government research funding	Opt in
The Aplastic Anaemia & Other Bone Marrow Failure Syndromes Registry	NS	2013	AUS	Clinician; Multicentre	NS	NS	Maddie Riewoldt's vision	Opt out
Haemoglobinopathy Registry	378 patients	2013	AUS	Clinician; Multicentre	Online	On funders request	Industry partners, Thalassaemia & Sickle Cell Australia, Thalassaemia Society of NSW	Opt out
Australian Bronchiectasis Registry	1053 patients (2018)	2015	AUS	Clinician; Multicentre	Web-based data entry	NS	National Health & Medical Research Council practitioner fellowship	Opt in
Monash Public Health Genomics*	NS	2015	AUS	Clinician; Multicentre	NS	NA	NA	NA
PLANET	NS	2019	AUS	Clinician; Multicentre	Online database /Mobile App	NS	Australian Government, Ipsen, Melbourne University	Opt in
Australian Cystic Fibrosis Data Registry	3538 patients	1996 (data collection in 1998)	AUS	Clinician; Multicentre	Online database	Annual reports & quarterly Newsletters	Cystic Fibrosis Australia, Vertex Pharmaceuticals	Opt in & Opt out
Australian Inherited Retinal Disease Registry	9298 patients (2020)	2009	AUS	Clinician; Multicentre	NS	Annual Reports	Retina Australia (WA)	Opt in
Australian Calciphylaxis Registry	47 patients (2014–2019)	2014	AUS	Clinician; Multicentre	Online database	Nil	Amgen Australia	Opt in
Bosentan Patient Registry	528 patients	2004–2007	AUS	Patient; Multicentre	NS	Nil	Actelion Pharmaceuticals	Opt in
The Australian Neuromuscular Disease Registry (covers Spinal muscular atrophy	448 patients (December, 2021)	NS	AUS	Patient; Multicentre	Online	Monthly & on demand	Biogen & Novartis, Philanthropic support Save our Sons & Muscular Dystrophy NSW	Opt in
Hypersomnolence Australia, Idiopathic Hypersomnia Patient Registry	NS	NS	AUS	Patient; Multicentre	Web-based data entry	Newsletter	Non-profit charitable organisation	Opt in
HHT Alliance (Hereditary Haemorrhagic Telangiectasia)	108 patients	2014	AUS	Clinician; Multicentre	Online database	NS	The Royal Melbourne Hospital Foundation	Opt in
Australian Mitochondrial Disease Foundation Patient Registry	486 patients (2020)	2014	AUS	Patient; Multicentre	Online form	Nil	The Mito Foundation, charity	Opt in
Leukodystrophy Australia	> 300 patients and families	NS	AUS	Patient; Multicentre	NS	Annual report	Australian Government’s Medical Research Fund	Opt in
Australian Autoinflammatory Diseases Registry	~ 200 people	2016	AUS	Clinician; Multicentre	REDCap database	On demand	Research grant	Opt in
Australian Bleeding Disorders Registry	3127 patients (2021)	1988	AUS	Industry; Multicentre	Paper form, mobile app, webpage	Annual report	National Blood Authority (NBA)	Opt in
Australian Motor Neurone Disease Registry	827 (in 2009)	2004	AUS	Clinician; Multicentre	NS	Nil	Charity—run by volunteers	Opt in & Opt out
The Rare Genetic Lipid Disorders Registry	16 patients (2019)	2019	AUS	Patient; Multicentre	Online database	NS	NS	Opt in
Myeloma Database in the Royal Adelaide Hospital, South Australia	743 patients	2009–2019	State	Clinician; Multicentre	NS	NS	NS	NS
South Australian Scleroderma Register	859 patients (2021)	1993	State	Industry; Multicentre	Online	Reported in other public reports	Australian Rheumatology Association	Informed consent
The Glomerular Disease Registry	120 patients (2020)	2018	State	Clinician; Multicentre	NS	Annual report	The George Institute for Global Health	Informed consent
Huntington's Disease Research Participant Registry	NS	2019	State	Patient; Multicentre	Online	NS	NS	Voluntary
Perth Demyelinating Diseases Database	983 patients (2009)	NS	State	Clinician; Single	Online	NS	NS	Informed consent
The South Australia Pathology iSYS database#	20 patients (2017)	NS	Umbrella	Industry; Single	Online	NS	NS	NS
Medical Genome Reference Bank*	4000 participants	2016	Umbrella	Industry; Multicentre	Web-based	NS	NS	NS
Australian Cancer Database#	353 cases with mucosal melanomas	1982	Umbrella	Industry; Multicentre	Online	NS	NS	NS
Australian Childhood Cancer Registry#	1269 patients (2020)	1983	Umbrella	Industry; Multicentre	NS	NS	NS	NS
Australian Paediatric Surveillance Unit Database#	1429	1993	Umbrella	Industry; Multicentre	APSU report card	NS	NS	NS
Queensland Oncology Online Database#	18 patients (2015–2017)	2005–2017	Umbrella	Industry; Single	Retrospective	NS	NS	NS
State-wide database based at Royal Perth Hospital, Western Australia#	526 patients (Feb 2015-Sept2017)	NS	Umbrella	Clinician; Single	Online database	NS	NS	NS
Gynaecological tumour registry of King George V Hospital	20 patients	1987–2000	Umbrella	Clinician; Single	Online database	NS	NS	NS
South Australian Clinical Cancer Registry#	140 patients	1980's	Umbrella	Industry; Multicentre	Online database	NS	NS	NS
South Australian Pathology Database#	NS	1960s	Umbrella	Industry	Online database	NS	NS	NS
The Victorian Cancer Council Registry#	251 patients (Jan 1998 to Dec2013)	NS	Umbrella	Industry	Online database	NS	NS	NS
Western Australia Register of Developmental Anomalies#	30,000 births annually	2011	Umbrella	Clinician	Online database	NS	NS	NS
Western Australia Cancer Registry#	95 patients (2021)	1981	Umbrella	Clinician	Electronic & paper records	NS	NS	NS
PMCC Prospective Database on Cervix Cancer Patients	NS	1998	Umbrella	Clinician	Online database	NS	NS	NS
University of Sydney Endocrine Surgical Unit Database	21 patients (Jul 1958 to Jun 2010)	1957	Umbrella	Clinician	Online database	NS	NS	NS

*ANZ* Australia & New Zealand; *APSU* Australian Paediatric Surveillance Unit; *AUS* Australia; *DIPG* diffuse intrinsic pontine glioma; *CDC* Centre for Disease Control and Prevention; *LGMD* limb girdle muscular dystrophy; *MCRI* Murdoch Children’s Research Institute; *NBA* National Blood Authority; *NHMRC* National Health and Medical Research Council; *NS* not stated; *NSW* New South Wales; *PMCC* Peter MacCallum Cancer Centre; SIOPE; European Society for Paediatric Oncology; *UK* United Kingdom; *WA* Western Australia

*Includes information of registries, databases and institutions collecting/researching people with rare disease conditions

#Information on State and Umbrella rare disease registries and databases listed in the table is relevant to specific disease or condition

Most of the registries, 47 (63.5%), mentioned in these studies, were established in the 2000s. The Neuroendocrine Tumour Registry (PLANET) [16, 17], Friedreich’s Ataxia Global Patient Registry [18] and Huntington’s Disease Research Participant Registry [19, 20] were established most recently, in 2019.

Forty-two (56.8%) registries used solely an online web-based data entry method. While only five (6.8%) registries use either, both paper and online web-based data entry or paper-based data entry only.

### Population size

The population size captured in each registry varied. At the time of this review, only 16 (21.6%) of the RDRs captured population of > 1000 patients. Five (6.8%) registries captured data of > 4000 patients. One registry, International Collaborative Gaucher Group (ICGG) Gaucher Disease Registry [21, 22], captured data of > 120,000 participants since it was established in 1994.

### Data management

Data management varied among the RDRs identified in this scoping review. Within the global registries, most of the data were entered by patients, their caregivers or other family members. Six global registries were clinician-initiated with the data entered by clinicians (ICGG Gaucher Disease Registry [22], the Global Retrospective Registry for Danon Disease [23, 24], the International and SIOP-E Diffuse Intrinsic Pontine Glioma (DIPG) Registries [25, 26], the International Dysferlinopathy Registry [27] and the International Schwannomatosis Database [28]).

In ANZ and Australian national registries, most registries collect their data online, with data entry completed by clinicians and registry staff. However, two of the Australian patient registries, Australian Bleeding Disorders Registry (ABDR) [29] and the PLANET registry [17], direct patients to enter their data via a mobile application.

Limited information was available on data management of the jurisdiction-based or “umbrella” registries identified in this scoping review.

### Data collected by the registries and databases

Most of the RDRs collected similar types of data, including patient demographics, clinical and diagnostic variables, procedure and treatment information, and PROMs.

Table 2 summarises data captured by RDRs and databases, described in this scoping literature review.

**Table 2:** Data captured by rare disease registries and databases

Registry name	Demographic variables	Diagnostic/clinical variables	Treatment/procedure details	QoL data	Timing of data collection
International Collaborative Gaucher Group Gaucher Registry	Gender, age	Diagnosis details, clinical, biochemical, & therapeutic characteristics regardless disease severity	Treatment status & choice, splenectomy status & date, treatment with alglucerase/imiglucerase	QoL questions—NS	(1) Baseline window: 12 months before to 1 month following date of imiglucerase initiation, (2) 10-year window: 8.5 to 11.5 years, (3) 20-year window: 17 to 23 years
Global Retrospective Registry for Danon Disease	Gender, age	Diagnostic details; genetic details; Baseline echocardiographic data	NS	NS	NS
Global Registry of Acute Coronary Events	Initials, DOB, address, telephone, physician, cardiologist details	Medical history, presentation symptoms, hospital dates, ECG findings, laboratory pathology results	Procedures, cardiac interventions, thrombolytics, medications, in-hospital events, discharge status	NS	6 months after discharge
International & SIOP-E Diffuse Intrinsic Pontine Glioma (DIPG) Registries	Country, gender, age	Diagnosis, date of diagnosis, imaging, signs & symptoms & physical exam at diagnosis, response evaluations, central pathology review characteristics, central imaging review characteristics, & molecular profile	Treatment data- chemotherapy, biopsies, pathology data	NS	Ongoing after registration
International Dysferlinopathy Registry	Basic demographics	Diagnosis details; symptoms &family history	NS	NS	NS
International Schwannomatosis Database	Contact details, DOB gender	Diagnosis details; symptoms & family history	NS	NS	NS
International Pachyonychia Congenita Research Registry	Name, surname, gender, DOB, address, doctor details	Age of diagnosis, disease characteristics, family characteristics	Images of disease areas, genetic testing details, medications	QOL symptom & pain questions	Once a year, target follow-up for 1 year
Wolfram Syndrome International Registry	Age, gender	Diagnosis details, family history	Genetic testing, DNA samples, & biological samples (blood & urine)	NS	NS
The Global Fukutin Related Protein Registry	Name, age, address, email, phone number	Diagnosis, motor function, pain, ventilation & family history, symptoms, lung, heart & cognitive function, contractures, six-minute walk distance & muscle strength	Medications, non-invasive/invasive ventilation use, brain/muscle MRI	NS	Annually
Cantú Syndrome Registry	Name, age, address, email, phone number	Medical history & health information, specific ABCC9 or KCNJ8 variant in the family (if any)	NS	NS	Annually
GNE Myopathy Registry	Age, 18+	Muscle Biopsy or genetic testing. General medical history; Level of physical activity	Medications	QoL questions	6 months, 12 months & Annually
Global Prader-Willi Syndrome Registry	Socioeconomic details, biological family history	Diagnostic details & medical history	Medications, supplements	Behaviour & mental health	As often as required
Global Angelman Syndrome Registry	Name, DOB, DOD, gender	Newborn & infancy history; history of diagnosis & results; Illnesses or medical problems; Behaviour & Development; Epilepsy history; Sleep Disturbance Scale for Children Pathology & Diagnostics	Medications & interventions	NS	Periodically
Idiopathic Thrombocytopenic Purpura Natural History Study Registry	Name, surname, age, gender, country, race, insurance, education, employment	Diagnostics tests, clinical visits, family history, & pregnancy/childbirth	Medications & frequencies/dosage, diet, surgeries	Enjoyment of life, fatigue, sleep, pain, social activities, mental health, physical health, PTSD symptoms, & financial impact	Within patient's own timeframe
Friedreich’s Ataxia Global Patient Registry	Name, DOB, gender	Diagnosis, medical history, functional mobility	NS	QoL—NS	1 month before anniversary, then annually
The Myotubular & Centronuclear Myopathy Patient Registry	Contact details & demographics; family history	Clinical diagnosis; genetic mutation; Genetic report & muscle biopsy report; Details of clinician & genetic testing/muscle biopsy centres	Best & current motor function & wheelchair use; Respiratory function, ventilation type & chest infections, Feeding & heart function; Neuromuscular examinations & scoliosis surgery	NS	NS
International Niemann-Pick Disease Registry	Individually identifying data: name, address	Diagnosis, health status, disease characteristics	Treatment, lab results, imaging tests	QoL questions—NS	Every six months
Limb-Girdle Muscular Dystrophy Type 2A Patient Registry	Name, DOB, gender, email, phone number, state, country, clinic name	Genetic diagnosis, genetic mutation, current ambulatory (walking) status, other family members with muscular dystrophy	Medications	NS	NS
International Fragile X Premutation Registry	Name, DOB, gender, contact details	Limited medical history	NS	NS	NS
Australian & New Zealand Fontan Registry	Height, Weight, BMI	Birth defects, congenital abnormalities, obstetric complications/interventions. cardiovascular health; condition specific information, general health, imaging, medication & supplements, reproductive health, linkage to other health databases	Re-intervention event(s), pacemaker status, cardiac transplant status, site of cardiac transplant procedure, Fontan take-down status, site of Fontan take-down procedure	PedsQL	NS
Thrombotic Microangiopathies Registry	Name, sex, DOB, doctor’s name	The circumstances and symptoms leading up to the diagnosis of TMA, other illnesses, medications, or history which could influence the onset & treatment of TMA	The type of treatment, the response of the illness to those treatments & any complications of the illness or treatment; relevant laboratory test & scan results	NS	NS
Australasian Epidermolysis Bullosa Registry	Age, sex, ethnicity, geographical & disease subtype distribution, past & current medical history, family history	Diagnostic laboratory studies, areas of blistering or erosion, EB type/subtype, electron microscopy, immunofluorescence, genetic mutation, complications (tracheolaryngeal, gastrointestinal, weight & growth development, ocular, genitourinary, skin cancers, renal disease, anaemia, swallowing problems) & clinical outcomes	Electron microscopy, immunofluorescence	NS	NS
Lymphoma & Related Diseases Registry	Demographics outcomes- overall & progression-free survival, duration of response & time to next treatment. Long-term outcomes- through linkage with cancer & death registries	Diagnoses, health status at diagnosis. Laboratory and imaging results at diagnosis, therapy, chemotherapy, autologous & allogeneic stem cell transplantation, & maintenance and supportive therapies: outcomes, long-term outcomes	Therapy	QoL	NS
Myeloma & Related Diseases Registry	General health diagnosis, gender, age	Diagnoses, clinical & laboratory results, therapy, decisions & complications, clinical outcomes	Autologous &allogeneic stem cell transplantation, therapy complications, laboratory results	EQ-5D-5L	NS
Australia & New Zealand Transplant Registry*	Name, gender, DOB, ethnic background, details of transfers between treating units	Baseline comorbidities, paediatric data; parenthood outcomes	Transplant Event, graft failure event	NS	Real time data collected on all new incident patients commencing RRT
Australian & New Zealand Registry of Advanced Glaucoma*	Fist name, surname, DOB, gender, address, phone number, email, eye clinician	Patient clinical details, diagnosis, BCVA, highest recorded IOP, refraction, central corneal thickness, cup disc ratio	Glaucoma surgery, laser trabeculoplasty	NS	NS
Australasian Interstitial Lung Disease Registry	Sex, age, ethnicity, mortality	Symptoms, clinical findings, occupational & environmental exposures, family history & co-morbid disease	Current & past medication lists (including oxygen use), results of other investigations including: serum blood markers, high resolution CT chest findings, blood gases, bronchoscopy +/− biopsy, active & past treatments specific to any form of ILD, adverse effects or incidents	Shortness of Breath Questionnaire, St George Respiratory Questionnaire & HADS	Baseline, Mortality data is reviewed every 6 months
The Australasian Registry Network of Orphan Lung Diseases	Reported disease, patient initials, DOB, postcode, medical record number, new case or follow up, physician details	NS	NS	NS	NS
Australia & New Zealand Familial Hypercholesterolaemia Registry	Personal & contact details, doctor’s details, relatives’ details for index patients, family pedigree, details on founder effect origin, family linkage details, clinical data at follow up assessments: hypertension, diabetes, antithrombotic, biochemistry profiles, & death	Family history, clinical history, physical examination, plasma LDL-cholesterol, biochemistry profile, risk factors and clinical trials, ‘Dutch Lipid Clinic Network Score’, ‘FH Diagnostic category’, ‘LDL-cholesterol adjusted for treatment’, ‘BMI’, genetic data (genotype & gene variants), laboratory reports	Medication details, drug intolerances, imaging tests: carotid ultrasonography, echocardiograms, coronary artery calcium scores, angiograms, & nuclear perfusion scans, e type, frequency, & complications	NS	NS
Australian National Creutzfeldt-Jakob Disease Registry	Full name, MRN, DOB, Address, doctor name, Hospital; Survival/mortality	Relevant medical conditions, including investigations, FNI outcome classification	Required to list any surgeries, transplants, grafts, blood donations/products or organs, dental/surgical care, renal dialysis, tattoos or piercings, or acupuncture	NS	From diagnosis—death
The Australian Rett Syndrome Database	Full name DOB, address, age at diagnosis, mothers age at subjects’ birth, mothers & fathers’ occupation, education, birth order	Genetic: MECP2 test, result & mutation identified, clinical severity	Treatment type dependant on symptoms	NS	Every 2 to 3 years
Lymphangioleiomyomatosis Registry	Full name, DOB, address, phone, email, gender, NOK	Date of diagnosis, spirometry and, when available, lung volumes and diffusing capacity), arterial blood gases or oximetry, walking and resting oxygen titration, cardiopulmonary stress testing, cause-specific mortality, functional status, & clinical events associated with lung transplantation	NS	NS	NS
Australian Genetic Heart Disease Registry	DOB, gender, ethnicity, COB, height & weight, family history	Clinical diagnosis, age at diagnosis, sudden cardiac death event, other serious medical conditions, symptoms, medications, genetic testing results, echo, ECG, cardiac MRI, exercise stress tests	Details of interventions, AICD indications, device details, adverse events & appropriate shocks. Use of services, cardiac investigations & clinical genetics consults	NS	Annually
Neonatal Alloimmune Thrombocytopenia Registry	Maternal demographics, neonatal demographics, postnatal features	Clinical case description, laboratory investigations (antibody investigations- immunofluorescence tests, glycoprotein binding ELISA assays & HLA antibody testing)	Transfusions, antenatal therapy, therapy & outcome, clinical outcomes	NS	Antenatal & Postnatal
Australian Idiopathic Pulmonary Fibrosis Registry	Demographics, smoking history, family history, medication history	Symptoms, objective investigational data relevant comorbidities (gastroesophageal reflux disease)	High-resolution computed tomography scans & any surgical lung biopsy specimens collected	QoL	Every 6 months
The Aplastic Anaemia & Other Bone Marrow Failure Syndromes Registry	Demographic details Clinical context, including possible precipitants, family history, including IBMFS, IPF & liver disease	Clinical presentation, laboratory test results at initial presentation & follow-up reviews	Immunosuppressive therapy, bone marrow or haematopoietic stem cell transplant, & supportive therapy; Clinical outcomes, including details of any relapse, complications, performance status indicators & disease progression	Nil	Data is collected through routine clinical visits
Haemoglobinopathy Registry	Full name, DOB, Medicare no, MRN, doctors’ details, ethnicity, gender	Height, weight, symptoms, diagnostic laboratory results, clinical &imaging results, complications of disease, family history	Therapy, clinical outcomes, transfusions,	NS	Multiple occasions (not specified)
Australian Bronchiectasis Registry	Full name, DOB, gender, Postal address, email address, COB	Age at diagnosis, diagnosis tests, clinical visits, comorbidities, genetic variations	Spirometry results, CT chest, radiology reports, airway cultures, medications	QoL-B	Baseline data, spirometry & CT details measured at enrolment, airway cultures obtained for 2 years prior to & 3mths after enrolment
Monash Public Health Genomics*	NA	The program conducts research centred around the genetic analyses of large cohorts, clinical trials and patient registries	Maintenance & supportive therapies;	NA	NA
PLANET	Full name, gender, DOB, clinician details	Bristol Stool Scale, Vital measurement (height & weight) ECOG scale—level of function	Compare types of treatments including; SSA PRRT, Chemo (Carbo/Etop), CAPTEM, Everolimus, Sunitinib, Telotristat & Immunolocy	QLQ-C30 & QLQGINET21	Periodically dependant on various treatments
Australian Cystic Fibrosis Data Registry	Name, Gender, DOB, COB, DOD	Diagnostic data; genetic mutation, initial diagnosis testing, clinical measures; lung function & BMI status, pulmonary infections & complications	Transplant history, hospital admissions, IVAb	Nil	Quarterly
Australian Inherited Retinal Disease Registry	Gender, DOB & contact details, ophthalmologist /referring doctor, family history & pedigree structure	Clinical data, results of electrophysiology tests, psychophysical measurement, ophthalmic examinations & genetic variations	Topical therapies, gene therapies, clinical trials	NS	NS
Australian Calciphylaxis Registry	Full name, DOB	Laboratory data, clinical background & presentation as well as therapeutic strategies & outcomes. Laboratory results	Treatment dialysis prescription, medications & treatment outcomes. Photos of skin lesions	Nil	Initial 5-year period of data collection, all treating units were contacted for patient follow-up data ongoing periodically, including calciphylaxis resolution & mortality
Bosentan Patient Registry	Name, gender, DOB	Health & vital statistics, clinical characteristics	Concomitant medications, length of treatment	Nil	At enrolment & at each 6-monthly visit
The Australian Neuromuscular Disease Registry	Full name, DOB, COB, gender, postal & email address	Genetic information, year of diagnosis, diagnostic tests, clinical visits, comorbidities	Clinical management, therapies, laboratory results, medications, frequency, dosages, surgical procedures	NS	6 & 12 months
Hypersomnolence Australia, Idiopathic Hypersomnia Patient Registry	Full name, DOB, email, postal address	Diagnosis, Multiple Sleep Latency Test results	Dose/type of medications, other medications, sleep apnoea status, treatment for sleep apnoea, other concerns	Nil	Once off
HHT Alliance (Hereditary Haemorrhagic Telangiectasia)	Full name, DOB address & Country of residence	Age & details of first diagnosis, physician details, family history of HHT	Surgical therapies, medical therapies, treatments manage complications	NS	Initial entry & subsequent update of available information as required
Australian Mitochondrial Disease Foundation Patient Registry	Full name DOB, DOD, gender, email & postal address	Clinical diagnosis, biochemical diagnosis, genetic diagnosis, type of mitochondrial disease	NS	Nil	6 & 12 months
Leukodystrophy Australia	Full name DOB, gender, email & postal address, COB	Year of diagnosis, diagnosis test, genetic results, family history of disease	Nil	Survival/mortality	Initial diagnosis, 6mths, 12 months & 24 months
Australian Autoinflammatory Diseases Registry	Full name, DOB, gender, email & postal address	Age at diagnosis, diagnostic testing, family history & genetic results	Clinical management, therapy, laboratory results	Nil	Once off—initial recruitment
Australian Bleeding Disorders Registry	Title, Full name, alias, DOB, address, phone email, doctor details	Date diagnosed, bleeding disorder, severity, baseline factor date/level, weight, height	Treatment regimen, product name, total dose, frequency	Nil	NS
Australian Motor Neurone Disease Registry	Patient demographics, site of onset of disease	Changes in disease, complications related to disease progression, diagnosis date	Treatment type & impact of new treatments & interventions	Nil	First presentation 3 months 6 months, approx. 12 months after your first visit, then ongoing every 6 months
The Rare Genetic Lipid Disorders Registry	Personal & contact details	Clinical history, family history, first physical examination. Genetic data & imaging, laboratory data	Management & treatment plans	Nil	Longitudinal follow up forms (not specified)
Myeloma Database in the Royal Adelaide Hospital, South Australia	Personal & contact details	Paraprotein subtype, cytogenic abnormalities at diagnosis, sites of involvement; biopsy-proven extramedullary myelomas involving skin, organ or CNS	Treatment details: autologous & allogenic stem cell transplants	NS	NS
South Australian Scleroderma Register	Demographics, sex, age at disease onset, residency & occupation at disease onset & 10 years before first symptoms, siblings, familial history of scleroderma, disease subtype	Incidence, clinical phenotype, possible precipitating factors, seasonality at presentation, presence of autoantibodies, survival; organ involvement, available serology	Immunofluorescence, counter immune-electrophoresis, enzyme-linked immunosorbent assay, line immunoblot	HAQ, SF-36, PROMIS-29 Profile 2.0, UCLA SCTC GIT 2.0, FACIT	Annual
The Glomerular Disease Registry	Basic demographics details	Medical history& details of disease diagnosis	DNA & blood samples	NS	Every 6 months through medical records & physician reviews
Huntington's Disease Research Participant Registry	Name, contact details, DOB, age, gender	Handedness (left or right h& dominant), description of health in terms of Huntington’s disease (diagnosis/genetics), general health-related questions	NS	NS	4 times a year
Perth Demyelinating Diseases Database	Gender, family history of MS, age at onset of initial symptoms, clinical course	EDSS score, MSSS score, VEP, disease duration	HLA-DRB1 genotyping, blood samples, MRI findings, cerebrospinal fluid studies	NS	NS

*AICD* automatic implantable cardioverter-defibrillator; *BCVA* best corrected visual acuity; *BMI* Body Mass Index; *CAPTEM* capecitabine and temozolomide; *CNS* Central Nervous System; *COB* Country of Birth; *CT* computed tomography; *DIPG* Diffuse Intrinsic Pontine Glioma; *DNA* deoxyribonucleic acid; *DOB* Date of Birth; *DOD* date of death; *EB* Epidermolysis Bullosa; *ECG* electrocardiogram; *ECOG* Eastern Cooperative Oncology Group; *ELISA* Enzyme linked Immunosorbent Assay; *EQ-5D* European Quality of Life Five Dimension; *FACIT* Functional Assessment of Chronic Illness Therapy; *FH* Familial Hypercholesterolaemia; *HADS* Hospital Anxiety & Depression Scale; *HAQ* Health Assessment Questionnaire; *HLA* Human Leukocyte Antigen; *HLA-DRB1* Major Histocompatibility Complex, Class II, DR Beta; *IBMFS* Inherited Bone Marrow Failure Syndromes, *ILD* Interstitial Lung Disease;*IOP* Intraocular pressure; *IVAb* Intravenous Antibiotics;*IPF* Idiopathic Lung Fibrosis; LDL; Low-density Lipoprotein; *MECP2* Methylcytosine-binding Protein 2; *MRI* Magnetic Resonance Imaging; *MRN* Medical Records Number; *MS* Multiple Sclerosis; *MSSS* Multiple Sclerosis Severity Scale; *NA* not applicable; *NS* Not Stated; *NOK* Next of Kin; PedsQL; Paediatric Quality of; PROMIS - 29 Profile 2.0: Patient-Reported Outcomes Measurement Information System - 29 Profile version 2.0; *PRRT* Peptide Receptor Radionuclide Therapy; *PTSD* Posttraumatic Stress Disorder; *QoL* quality of life; *QoL-B* quality of life questionnaire; *QLQ-C30* European Organisation for Research and Treatment of Cancer. Core Quality of Life Questionnaire; *QLQGINET21* Questionnaire for Patients with Gastrointestinal Neuroendocrine Tumours; *RRT* renal replacement Therapy; SIOPE; European Society for Paediatric Oncology; *SSA* Somatostatin Analogues; *SF 36* Short Form 36; *TMA* Thrombotic Microangiopathies; *UCLA SCTC GIT* University of California Los Angeles Scleroderma Clinical Trial Consortium Gastrointestinal Tract; *VEP* visual evoked potential

#### Demographic data

Demographic variables included given names, surnames, age, gender, country, address, email, phone number, death date, race/ethnicity, insurance, education, employment status, family history, next of kin, clinic and doctor’s details. The information was anonymised for most RDRs and databases. Individually identifying data was only collected in the International Niemann-Pick Disease Registry [30, 31]. As the development of the International Niemann-Pick Disease Registry is ongoing, the use of identified data will aid in the registries planning for linkages to clinical and patient datasets.

#### Diagnostic/clinical data variables

Diagnostic/clinical data variables generally included some or all of the following: (1) the presence of disease symptoms and clinical examination findings; (2) investigation findings such as pathology investigation and biopsy/cytology results, medical imaging investigations, genetic profile, and resulting date of diagnosis; (3) functional and behavioural status and support needs related to the disease; and (4) the presence of disease sequelae, including morbidity and death.

For paediatric RDs, a broader paediatric history was also often included (Table 2). Some clinical and diagnostic information was unique to each RDR. For example, the ICGG Gaucher Disease Registry [22, 32] collected additional details on biochemical and therapeutic characteristics, while the DIPG registry [25] captured symptom duration, cranial neve palsy, pyramidal signs, cerebellar signs, tumour material, imaging details, signs and symptoms and physical exam at diagnosis, response evaluations, central pathology review characteristics, and molecular profile. The Australian Bronchiectasis Registry records spirometry results, computerized tomography (CT) chest scans, radiology reports, airway cultures and pulmonary exacerbations as their baseline data [33, 34], while the Neonatal Alloimmune Thrombocytopenia (NAIT) records maternal and paternal testing result details [35, 36].

#### Treatment/procedure details

Disease management information included: (1) medical management, including medications; (2) surgical and procedural information; (3) exacerbation/critical care episodes; and (4) supportive care, such as nutritional and allied health interventions. This information was similar across all the registries; however, additional information was available for individual RDRs. Only three ANZ RDRs collected surgical procedures and treatment information.

#### PROMs and QoL data

PROMs and QoL data were captured by five global registries (ICGG [22], International Pachyonychia Congenita Research Registry [37, 38], *GNE* Myopathy Registry [39], Friedreich’s Ataxia Global Patient Registry [18], Immune Thrombocytopenia (ITP) Natural History Study Registry [40]), which captured physical and mental health, post-traumatic stress disorder symptoms, social activities and financial impact.

Only four ANZ RDRs captured PROMs and QoL information. These include the ANZ Fontan Registry [41], Myeloma and Related Diseases Registry (MRDR) [42], Lymphoma and Related Diseases Registry (LARDR) [43] and the Australasian Interstitial Lung Disease Registry (AILDR) [44]. Amongst the Australian RDRs, 24 registries collected PROMs. Similar to most RDRs discussed in this review, the specific PROMs collected varied depending on the registry.

#### Timing of data collection

Most global RDRs and databases captured data annually; however, some had specific data entry requirements. For example, the ICGG Gaucher Disease Registry collected data at multiple time points: (1) baseline (2 months before to 1 month following the date of imiglucerase initiation), (2) 10-year window (8.5–11.5 years), and 3) 20-year window (17–23 years) [22]. In the Global Angelman Syndrome registry participants may receive a request to update data periodically [45, 46]. The *GNE* Myopathy Registry [39] contacted their patients at 6 months and yearly. Friedreich’s Ataxia Global Patient Registry [18], Cantú Syndrome Registry [47, 48] and the International Pachyonychia Congenita Research Registry [37] collected data annually.

While there was limited data available on the timing of data collection throughout the ANZ RDRs, it was noted that the ANZDATA registry distributes an annual survey for all dialysis and transplant units in ANZ on the 31st of December [49]. The AILDR data are collected at baseline, and mortality data are reviewed every 6 months with dates of death and lung transplantation recorded as determined by clinical records and/or death certificates [44]. Families and carers in the Australian Rett Syndrome Database are invited to contribute information every 2–3 years about changes in the health or function of the individual with Rett syndrome for whom they care [50]. To assess patient outcomes in the Australian Calciphylaxis Registry (ACR) [51], following the initial 5-year period of data collection, all treating units are contacted for patient follow-up data, including calciphylaxis resolution and mortality. Other registries and databases capture data annually.

#### Registry reporting and funding

Annual reports, newsletters and research publications were the main reporting outputs for 32 (43%) RDRs identified in this review (Table 2). Funding the operations of the registries and databases varied from industry partners, non-profit charity organisations and fundraisers and government grants.

## Discussion

Registries are often established to describe patterns of care, and to understand variation in treatments and outcomes and predictors of prognosis and QoL. Establishing and maintaining a registry requires substantial resources, infrastructure and sustained funding [52].

The present scoping review examined publicly-available data on existing RDRs and databases that included Australian participants. The findings of this review are an important first step towards informing the development of a national framework for the RDRs in Australia.

Seventy-four different registries and databases of RDs collecting Australian data were identified; 19 of them were global registries, 24 were Australian-only registries, 10 were ANZ based, and five Australian jurisdiction-based registries. Sixteen “umbrella” registries collected data on several rare conditions, which included some RDs, and thirteen registries stored rare cancer-specific information.

The population size captured in each registry varied, with many being relatively small. This highlights the challenges associated with diagnosis and reporting, as few clinicians have experience in managing individuals with RDs. Lack of clinician awareness can lead to significant delays in diagnosis or individuals remaining undiagnosed, creating future RD data deficits [53].

Data management and entry varied among the RDRs, with data either being entered by patients/caregivers/family members, clinicians or by the registry staff. Most of the Australian-only RDRs identified in this review were clinician-led. For ultra RDs where patients are few, building a patient registry is an intuitive first step to determining the number of people affected, their geographical distribution and basic demographic and clinical characteristics of the disease. The scope of these registries may evolve over time, maturing from a community effort as a means for a basic understanding of patient and disease characteristics, to a supportive mechanism for research funding and attracting input from health service providers to registry data collection [54].

Identifying a common minimum data set for RDRs is a challenge due to the heterogeneous nature of RDs. The RDR data sets identified in our review generally comprised a variety of data elements within the domains of demographic; diagnostic/clinical; treatment/procedure and PROMs and QoL data, with individual variables being unique to RDs. In 2011, the European Commission funded the EPIRARE project (‘Building Consensus and Synergies for the European Union (EU) Registration of Rare Disease Patients’), prior to the establishment of a European Platform for RDRs, which aims to support a harmonized approach to European RDRs [55–57]. A ‘set of common data elements for Rare Diseases Registration’ was produced by a Working Group coordinated by the Joint Research Centre and composed of experts from EU projects: EUCERD Joint Action, EPIRARE and RD-Connect.

The European Platform on RD Registration (EU RD Platform; European Commission) identified 16 common data elements for the initial registration of people with RDs onto a RDR. These are grouped in the following categories:

1. Pseudonym.
2. Personal information (date-of-birth, sex).
3. Patient status (alive, dead, long-term follow-up, opted out).
4. Care pathway (date of initial specialist contact).
5. Disease history (Age at symptom onset; age at diagnosis).
6. Diagnosis (Disease diagnosis, Genetic diagnosis, undiagnosed).
7. Research (contact for research, data for secondary use, biobank).
8. Disability (functioning, disability).

While a detailed mapping of each Australian registry’s dataset against elements was beyond the remit of this scoping review, a majority of identified RDRs collected items that aligned with the broad categories recommended by EPIRARE [58]. In addition, a number of RDRs in Australia collected follow-up clinical and patient-reported information.

PROMs and QoL data were captured only by some of the RDRs identified in our review. PROMs are increasingly being introduced into clinical registries in Australia, providing a person-centred perspective on the expectations and impact of treatment [59]. Including PROMs in clinical registries offers numerous advantages [60]. First, incorporation of the patient voice helps keep outcome measurements of care person-centred. Further, symptom burden, QoL and satisfaction with care are dynamic variables that cannot be recreated accurately through retrospection; they are essentially lost if not captured “in the moment”. PROMs data collection has also been supported by the EPIRARE [58].

Frequency of data collection was not consistent throughout the RDRs captured in this review. Determining regular intervals and time points for data capture in RDRs can be challenging due to low patient numbers and frequent loss to follow up. Overtime, retention of patients and providers can also be difficult, so registry developers should build in mechanisms for monitoring and follow-up [61].

RDR reporting information was not available for more than half of the RDRs identified in this review. This could be because these RDRs were not established with a purpose for quality improvement as an outcome [62]. This is a noteworthy limitation of many RDRs in Australia, that should be addressed by a more coordinated national approach, including alignment with the recently published National Strategy for Clinical Quality Registries and Virtual Registries [63].

Our review identified opportunities to learn from successful RDRs, as well as from those that have not been sustained. Established in 2009, the Australasian Registry Network of Orphan Lung Diseases (ARNOLD) is one example of an unsustainable registry [64]. ARNOLD aimed to provide prevalence data for multiple orphan lung diseases in both the paediatric and adult populations [64]. During its operation ARNOLD obtained national data relating to 30 rare and extremely rare lung diseases. However, the registry faced several barriers. Data were limited by the under-reporting of patient identifiers and other details. Only 35% of notifications to the registry included postcode, birthdate or patient initials, so duplicate notifications could not be identified, which may have led to over-reporting of some rare conditions. Another limitation of this registry was specific diseases were not well defined, so reporting relied on individual physician diagnoses, which may have led to inconsistencies. Nonetheless, the registry operators noted that many physicians recognised the importance of contributing to a web-based RDR to monitor quality of care [64].

An exemplar RDR is the Australian Cystic Fibrosis Data Registry (ACFDR) [65, 66]. The ACFDR has been funded, from its commencement, by Cystic Fibrosis Australia through a combination of fundraising, philanthropy and industry support. The ACFDR collects information from people with cystic fibrosis from the time of diagnosis to the time of death or lung transplantation. Data is entered into the registry by clinicians and data managers from 23 public centres. Data completeness has been enhanced over the last few years through an industry-sponsored data quality program, leading to increased acceptability of the data and increasing use for research and quality improvement. The success of the ACFDR is attributed to sustained funding over a prolonged period, broad clinical and community support, and experienced registry management.

The concept of a single national RDR or database in Australia is worth consideration and has been suggested previously [1]. National RDRs have been also established in other countries, including China [67], Italy [68] and other European countries [69]. In 2019, researchers in Slovenia developed a national RDR pilot informed by focus groups with experts from leading institutions in the field of RD [70]. The results indicated that effective development of a national RDR requires a series of systemic changes and many considerations [70].

The European Society for Immunodeficiencies (ESID) Registry was utilised as a platform for the German Network on Primary Immunodeficiency Diseases (PID-NET) to perform queries on RDRs and extract the data in the context of PID-NET [71, 72]. To interconnect RDRs the Open Source Registry System was implemented, based on a federated search functionality, ultimately making data from the ESID registry available in an interoperable manner and without losing sovereignty [73].

For several years, there have been activities ongoing in Germany under the Medical Informatics Initiative to digitally bring the hospitals together, combining the development of IT infrastructure, scientific research projects, as well as the promotion of junior researchers and education in medical informatics [74]. This initiative has been realized within five consortia of which two are mainly using Open-Source software [75], namely the consortia MIRACUM [76] and DiFUTURE [77]. They work on distributed data analysis using Data Shield [78] and the Personal Health Train [79], the latter designed for distributed machine learning. A combination of the aforementioned methods could potentially lead to a robust solution allowing RD data to remain within the registries without creating a new umbrella RDR, to use attachable structure leveraged by established tools, in order to interconnect RDRs nationally and internationally.

To achieve this in Australia, well-coordinated and well-funded efforts should involve all stakeholders, as well as alignment of all medical, organisational and technological aspects in accordance with the long-term public healthcare objectives and infrastructure for clinical trials. With differing perspectives on key issues related to RDRs, such as their sustainability, data custodianship, data entry requirements, and other challenges including limited patient numbers, a lack of infrastructure and workforce, funding sources and governance structures, the development of formal policies to support coordination and sustainability of Australian RDRs has been complex and difficult.

Such initiatives require behavioural, regulatory, financial, organizational, process, policy, clinical, and many other changes at all levels of the healthcare system. Dedicated funding should be allocated for eligible RDRs to support their reach to recruit every possible consenting patient across Australia and ensure full coverage of the eligible clinical population. Incentives for hospital and health services should encourage staff to continue dedicating their time to RD data collection. Investing in the infrastructure and staffing would assist in streamlining and simplifying the maintenance and data entry demands of new and existing RDRs across Australia. Finally, RDRs should be promoted in hospitals and relevant clinics, and collaborations with relevant international registries should be sought [13]. As we take steps towards a nationally coordinated approach we should leverage data and learnings from existing RDR captured in this review. Information regarding RDRs operating in Australia could be consolidated into a repository to support the development of new RDRs. Aligning with the previous work of EPIRARE and the EU RD Platform in defining a minimum data set, a national set of best practice principles for RDRs in Australia could be developed, using the ACFDR as an exemplar. This could include principles of success derived from this review, as well as individual RDR experiences, including:

- Clinician-led, person-centred governance.
- Low burden approvals.
- Ongoing sustainable funding.
- Low burden data collection & linkage with existing data sources.
- Mechanisms for consumer contribution.
- Prospective, incidence-focused registries that address knowledge gaps.
- Enablement of multiple data uses, with consumer opt-out provisions.
- Broad data uses, including for reporting, epidemiology, secondary research, access to clinical trials.
- Support consumer connection and mechanisms to report back to registry participants.
- Data interoperability/harmonization—data sharing/linkage between datasets/registries.

Research and data are one of the three key pillars in the Action Plan [13]. Limited data is a common feature of RDs, often resulting in high uncertainty, which impacts every part of people’s lives. People are faced with impossible choices based on incomplete knowledge and unclear pathways. In development of the Action Plan the sector highlighted comprehensive, high quality collection, and effective use of RD data as one of four critical enablers of effective RD policy [13]. RDRs have unique advantages that can be leveraged for success. These advantages include engaged communities who value data and knowledge and are highly attuned to the benefits of RDRs in facilitating access to participation in research and clinical trials. In addition, the benefits of novel therapies and precision medicine for RDs are likely to encourage the establishment of future RDRs for the support of post-marketing surveillance.

### Strengths and limitations

This study has systematically and comprehensively reviewed publicly-reported data collected by RDRs with Australian participants, as reported in the literature. To appreciate the findings in this review, the following limitations should be considered. First, we have likely missed several RDRs and databases that collect RD information because a lot of data is kept in secure electronic health records despite our comprehensive search strategy, including an internet search in addition to a literature search of four large electronic databases. Second, most publications or grey literature sources did not provide detailed information on the data items captured by RDRs and databases. For state-wide and “umbrella” registries, the information and numbers reported are accurate at the date of publication of the source and for the particular condition, as these registries may also capture other RDs and conditions. We have identified a few state/jurisdiction and “umbrella” registries that capture data from various conditions, including RDs. Registry information is relevant to the conditions only listed in the tables. This has been noted in the legends and in the text. Since we focussed on the registries collecting data from people with RDs, we are aware that not all RDs captured by the state and “umbrella” registries may be described in this scoping review. Third, RDR literature did not usually report on the impact of registries on patient outcomes, nor did they detail enablers and barriers to meeting registry objectives. This requires further investigation and follow-up with registry managers.

## Conclusion

The findings of this scoping review highlight the heterogeneity of RDRs collecting Australian data, with variations noted in the mechanisms for data entry, data items captured, scope, outputs, and sources of funding. This heterogeneity calls attention to the challenges of establishing and maintaining RDRs in Australia and the interest in a nationally consistent approach, as highlighted in the Action Plan [13]. Nevertheless, the different characteristics and needs of individual RDs in Australia make interoperability difficult, especially given the absence of a nationally consistent minimum dataset for RDs.

An initial step would be to consolidate information regarding existing RDRs to support the development of new registries. This should be followed by development of Australian RDRs best practice consensus guidelines, aligned with existing international standards, with goals to improve clinical practice and outcomes, access to research and clinical trials, and support the RD community. Investment in a nationally coordinated and consistent approach to data collection for RD in Australia will improve our understanding and quantification of the national burden of RDs, and their impact on patients, carers and families. A national strategy for RD data collection will also require cross-jurisdictional government engagement and agreement. This requires systemic policy reform, but is a critical way for governments to keep pace with international direction and respond to the Action Plan for the best possible health and wellbeing outcomes for Australians living with a RD.

Few of the seventy-four RDRs identified in this review were sustainable funded or reported regular research and quality improvement outputs. This suggests an under-investment in RDRs in Australia compared to more common conditions—diabetes and obesity, heart disease and cancer, which have been prioritised as areas for CQRs by the Australian Commission for Safety and Quality in Health Care [80]. Not many RDRs identified in this review outline a clear purpose or scope, and the data they collect is often incomplete and varied. These factors are contributing to poor data quality and poor levels of clinical awareness and knowledge. To achieve equity in data and knowledge of RD that is aligned with more common conditions, investment in RDRs is vital. Without such investment rare diseases cannot be counted and evidence-based improvements in care and outcomes will not keep pace with rapid advances in medical technologies, particularly in the areas of genomics and precision medicine.

## Data Availability

Not applicable.

## Abbreviations

ACFDR: Australian Cystic Fibrosis Data Registry
ABDR: Australian Bleeding Disorders Registry
ACR: Australian Calciphylaxis Registry
AILDR: Australasian Interstitial Lung Disease Registry
ANZ: Australian and New Zealand
ARNOLD: Australasian Registry Network of Orphan Lung Diseases
CT: Computerized tomography
DIPG: Diffuse Intrinsic Pontine Glioma
ESID: European Society for Immunodeficiencies Registry
EU: European Union
ICGG: International Collaborative Gaucher Group
ITP: Immune Thrombocytopenia
LARDR: Lymphoma and Related Diseases Registry
MeSH: Medical Subject Heading
MRDR: Myeloma and Related Diseases Registry
NAIT: Neonatal Alloimmune Thrombocytopenia
PID-NET: German Network on Primary Immunodeficiency Diseases
PRISMA-ScR: Preferred Reporting Items for Systematic Reviews and Meta-Analyses extension for scoping reviews
PROMs: Patient-Reported Outcome Measures
QoL: Quality of life
RD: Rare disease
RDR: Rare disease registry
RVA: Rare Voices Australia
UK: United Kingdom
US: United States
